# Association Between Metabolic Syndrome and an Increased Risk of Hospitalization for Heart Failure in Population of HFpEF

**DOI:** 10.3389/fcvm.2021.698117

**Published:** 2021-09-14

**Authors:** Ying Zhou, Liyao Fu, Jiaxing Sun, Zhaowei Zhu, Zhenhua Xing, Shenghua Zhou, Shi Tai, Yongjun Wang

**Affiliations:** ^1^Department of Blood Transfusion, The Second Xiangya Hospital of Central South University, Changsha, China; ^2^Department of Cardiovascular Medicine, The Second Xiangya Hospital of Central South University, Changsha, China

**Keywords:** heart failure with preserved ejection fraction, metabolic syndrome, cardiovascular disease, cardiovascular mortality, hazard ratios

## Abstract

**Background:** The association between metabolic syndrome and the development of heart failure (HF) with preserved ejection fraction (HFpEF) has not been completely clarified.

**Aim:** To evaluate the association between metabolic syndrome and the risk of HF hospitalization for patients with HFpEF.

**Methods:** Patient data were obtained from the American cohort of the Treatment of Preserved Cardiac Function Heart Failure with an Aldosterone Antagonist (TOPCAT) trial database. Data for the primary outcome (hospitalization for HF) and secondary outcomes (all-cause mortality, cardiovascular mortality, and all-cause hospitalization) were collected, and hazard ratios (HRs) for the patients with and without metabolic syndrome were analyzed by applying a multivariable Cox proportional hazard model.

**Results:** Among the 1,548 total participants, 1,197 had metabolic syndrome. The patients with metabolic syndrome exhibited worse heart function and a lower quality of life than those without metabolic syndrome. During the 3.3 years of follow-up, 351 patients were hospitalized for HF. After a multivariable adjustment, the risk of hospitalization for HF and all-cause hospitalization (adjusted HR = 1.42, 95% CI: 1.01–2.00; *p* = 0.042 and adjusted HR = 1.27; 95% CI: 1.04–1.54; *p* = 0.017, respectively) were independently associated with HFpEF for the patients with metabolic syndrome. In addition, the risks of HF hospitalization and all-cause hospitalization among 267 propensity score-matched patients were higher for patients with metabolic syndrome (HR = 1.53, 95% CI = 1.05–2.23, and *p* = 0.025 and HR = 1.34, 95% CI = 1.08–1.67, and *p* = 0.009, respectively).

**Conclusion:** The risks of HF hospitalization and all-cause hospitalization were higher for patients with HFpEF having metabolic syndrome than for those without metabolic syndrome.

## Introduction

Heart failure (HF) with preserved ejection fraction (HFpEF) is an increasingly serious global health problem that occurs in half of the patients hospitalized with HF (1). HF syndrome and a normal left ventricular ejection fraction (LVEF) are the main clinical features of HFpEF (2), whereas the fundamental pathophysiology of HFpEF is poorly understood, there is a growing recognition that it is a heterogeneous syndrome. The complex clinical manifestations that characterize HFpEF are aggravated due to the presence of multiple comorbidities, including metabolic syndrome (2, 3).

Metabolic syndrome has a significantly negative impact on HF and its prognosis (4). In a Japanese HF cohort, the prevalence of metabolic syndrome in the patients with HF was more than double that of the general population (5). The Uppsala Longitudinal Study of Adult Men (6) found that the risk of incident HF was 5.3 per 1,000 person-years for subjects with metabolic syndrome vs. 1.7 per 1,000 person-years for those without metabolic syndrome, thus confirming that metabolic syndrome is a good predictor of HF. Metabolic syndrome increased the risk of developing HF by more than 3-fold, and this increased risk remained when adjustments were made for established risk factors for HF. In addition, a 20-year follow-up study of elderly Finns demonstrated that metabolic syndrome could predict incident congestive HF (7).

However, studies on the outcomes and characteristics of patients with HFpEF and metabolic syndrome are limited, and it remains unknown whether the metabolic syndrome is associated with worsening HFpEF. To study potential phenotype-specific targets to guide clinical practice, it is necessary to clearly understand the clinical characteristics and prognostic features of patients with HFpEF having metabolic syndrome. Therefore, the current study aimed to evaluate the association between metabolic syndrome and the risk of hospitalization for the management of HF (subsequently referred to as HF hospitalization).

## Materials and Methods

### Patients

**Figure 1:**
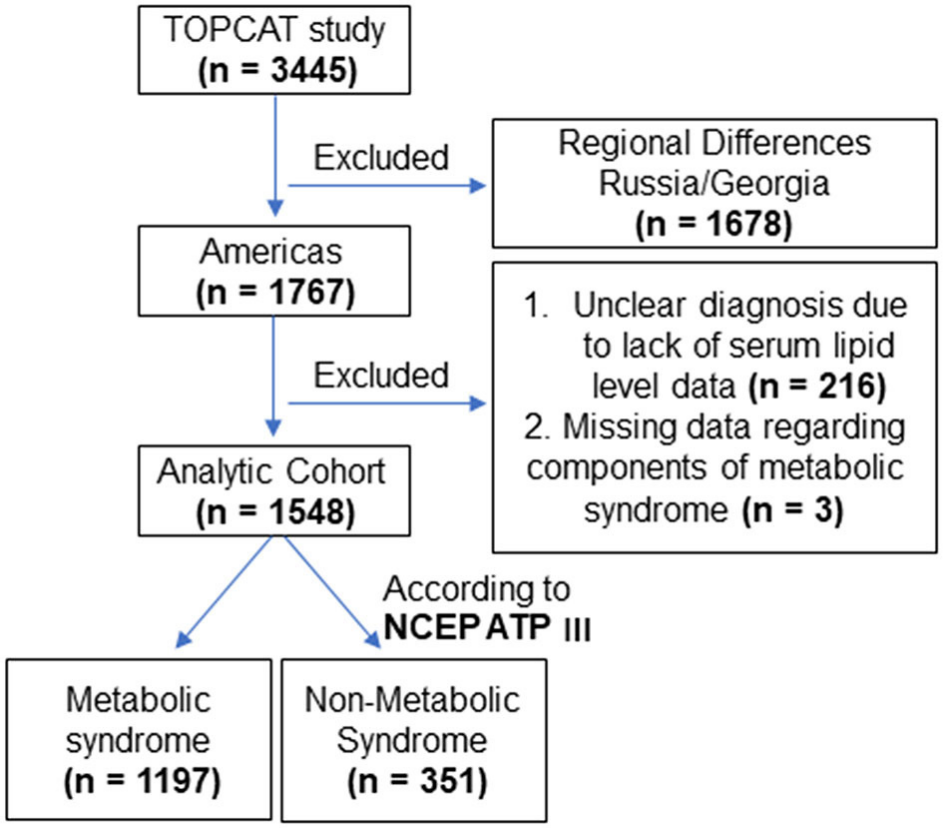
The enrollment chart, which shows the patients that were included in the final sample; TOPCAT: Treatment of Preserved Cardiac Function Heart Failure with an Aldosterone Antagonist trial; NCEP ATP III: metabolic syndrome diagnosis based on the working definition proposed by the National Cholesterol Education Program (NCEP) Adult Treatment Panel (ATP) III.

This study is based on a publicly available, de-identified version of a database of patients collected from the randomized, placebo-controlled Treatment of Preserved Cardiac Function Heart Failure with an Aldosterone Antagonist (TOPCAT) trial, which has been released by the National Heart, Lung, and Blood Institute's Biologic Specimen and Data Repository Information Coordinating Center (BioLINCC; https://biolincc.nhlbi.nih.gov/studies/topcat/). The patients in the TOPCAT trial were enrolled at 233 centers across the United States, Canada, Brazil, Argentina, Russia, and Georgia from August 2006 to January 2012. The study design, detailed protocol, and patient characteristics have been reported previously (8). A total of 3,445 patients with HFpEF in the TOPCAT trial were included in the current study according to the following criteria: (1) ≥ 50 years of age, (2) at least one sign and symptom of HF, (3) an LVEF ≥ 45%, (4) controlled systolic blood pressure, (5) a serum potassium level <5 mmol/l, and (6) a brain natriuretic peptide (BNP) level ≥100 pg/ml or an N-terminal pro-BNP level ≥360 pg/ml within 60 days before randomization due to a history of HF hospitalization within the previous 12 months. The exclusion criteria included a life expectancy <3 years caused by severe systemic illness with severe renal dysfunction and specific coexisting conditions, medications, or acute events. The eligible participants were randomly assigned to receive either spironolactone or a placebo. The mean follow-up duration was 3.3 years. Since there was uncertainty about whether the TOPCAT participants from Russia and Georgia had HF, the final analysis was limited to the 1,767 participants from America, as shown in Figure 1. The rationale, design, and main findings of this trial have been published previously (8).

### Data Collection

All the demographic data and laboratory examination indicators for the patients were collected from the trial database stored at BioLINCC. The primary outcome (hospitalization for HF) and secondary outcomes (all-cause mortality, cardiovascular (CV) mortality, and all-cause hospitalization) were recorded and assessed separately. CV mortality included death from myocardial infarction (MI), stroke, sudden death, pulmonary embolism, pump failure, and CV procedure-related events. All occurrences of the individual components of the primary outcome, and those of MIs and strokes, were adjudicated by a clinical end-point committee at Brigham and Women's Hospital (Boston, MA, USA) according to prespecified criteria. Additional details regarding the evaluation of the outcomes have been reported previously (8).

### Definitions

In the present study, metabolic syndrome was diagnosed based on a working definition proposed by the National Cholesterol Education Program (NCEP) Adult Treatment Panel (ATP) III. Subjects with three or more of the following criteria were classified as having metabolic syndrome according to the NCEP ATP III report (9): (1) fasting glucose >100 mg/dl (5.6 mmol/l); (2) diastolic blood pressure >85 mmHg or systolic blood pressure >130 mmHg; (3) triglyceride concentration >150 mg/dl (1.7 mmol/l); (4) high-density lipoprotein cholesterol level <40 mg/dl (1.02 mmol/l) for men and <50 mg/dl (1.29 mmol/l) for women; and (5) abdominal obesity, as defined by a waist circumference >102 cm for men and >88 cm for women. In addition, individuals who were currently taking drugs to treat diabetes (insulin or oral agents) were considered to have diabetes, and those taking drugs to control hypertension or dyslipidemia were considered to have high blood pressure or dyslipidemia, respectively. Among the 1,767 original patients, those with missing metabolic syndrome component data or an unclear diagnosis because of lacking raw data were excluded from the study (*n* = 219). Finally, we enrolled 1,197 patients with HFpEF having metabolic syndrome and 351 patients with HFpEF and without metabolic syndrome (Figure 1).

Echocardiography was performed for 565 of the patients. The evaluation of diastolic dysfunction was based on the mitral inflow E/A ratio, tissue Doppler E′, and deceleration time (10), and it was graded as follows: mild, a reduced E′ (septal < 8 cm/s or lateral < 10 cm/s) and an E/A ratio ≤0.8; moderate, a reduced E′ and an E/A ratio of 0.8–1.5; and severe, a reduced E′ and an E/A ratio > 1.5 or an E-wave deceleration time <160 ms. Diastolic dysfunction was graded only among the participants who were in sinus rhythm. The health status of the patients with HF was assessed using the Kansas City Cardiomyopathy Questionnaire (KCCQ), which includes questions regarding physical limitations, symptoms (frequency, severity, and recent changes over time), social interference, self-efficacy, and quality of life. The KCCQ scores were converted to a value between 0 and 100, with higher scores indicating a better HF-specific health status. The EuroQOL Health Status Questionnaire (EQ-5D) was also used to assess the health status of the patients, and it was evaluated using a visual analog scale (0–100, with the worst state indicated by a score of 0 and the best state indicated by a score of 100). Finally, the Patient Health Questionnaire (PHQ) was used to assess the depression status of patients [no depression (0–9) vs. depression (10+)].

### Statistical Analysis

The qualitative demographic data are presented as numbers (%), and the baseline characteristics of the patients with and without metabolic syndrome were compared using a chi-square test. The quantitative data are presented as the mean ± SD, and Student's *t*-test was used to compare the baseline characteristics.

The Kaplan–Meier survival curves were used to analyze the primary and secondary outcomes for the patients with and without metabolic syndrome, and the differences between the groups in the cumulative incidence curves were compared using a log-rank test. In addition, a Cox proportional hazards regression analysis was performed to calculate the hazard ratios (HRs) for the primary and secondary outcomes with 95% CIs. The proportional hazards assumption was examined with Schoenfeld residuals. Multivariable adjustments included *a priori* selected variables, and we used three models. Model 1 included sex, age, race, alcohol consumption, and smoking status. In model 2, we added New York Heart Association (NYHA) functional class, MI, angina pectoris, atrial fibrillation (AF), stroke, peripheral arterial disease, implanted cardioverter defibrillator, implanted pacemaker, chronic obstructive pulmonary disease, treatment using coronary artery bypass grafting (CABG) or percutaneous coronary intervention (PCI), estimated glomerular filtration rate (eGFR), blood urea nitrogen, hematocrit, potassium, chlorine, total bilirubin, and randomization arm (spironolactone or placebo). Model 3 included the addition of a calcium channel blocker (CCB), an angiotensin II receptor blocker (ARB), or an angiotensin-converting enzyme inhibitor (ACEI), a beta-blocker, or a diuretic.

The primary and secondary outcomes for the propensity score-matched patients with and without metabolic syndrome were assessed using a Cox proportional hazard analysis. We used 1:1 nearest-neighbor matching without replacement to match all the baseline characteristics (except for the components of metabolic syndrome). The propensity score was derived using a logistic regression model that included metabolic syndrome as the outcome variable and various potential confounders as explanatory variables. Standardized differences of <0.10 between the propensity score-matched patients were considered negligible. To explore effect modification, we tested the interaction between abdominal obesity (a component of metabolic syndrome) and obesity [defined as body mass index (BMI) ≥ 30 kg/m^2^] to predict future HF hospitalization, which was further analyzed using the following subgroup analyses: obesity (non-obesity vs. obesity), diabetes (non-diabetes vs. diabetes), age (<70 vs. ≥70 years), sex (male vs. female), MI (non-MI vs. MI), AF (no history of AF vs. history of AF), NYHA functional class (NYHA I and II vs. NYHA III and IV), and treatment arm (spironolactone vs. placebo). Additionally, as above, interactions between metabolic syndrome and the subgroups were tested, with respect to future HF, to explore effect modification in a multivariable model. A two-sided *p* < 0.05 was considered significant. All the statistical analyses were performed using Statistical Product and Service Solution version 25 (IBM, Armonk, NY, USA).

## Results

### Patient Characteristics

Of the 1,548 patients analyzed, 1,197 (77.3%) had metabolic syndrome and 351 (22.7%) did not have metabolic syndrome. The baseline characteristics of patients are shown in Table 1. The patients with HFpEF having metabolic syndrome were younger and had more comorbidities, including coronary artery disease and interventions (MI, PCI, CABG, and angina pectoris), peripheral arterial disease, and previous stroke, than those without metabolic syndrome. In contrast, AF was more prevalent in patients without metabolic syndrome. The patients with metabolic syndrome had significantly lower eGFR, hemoglobin, hematocrit, and total bilirubin values, but their blood urea nitrogen value was higher; they also demonstrated higher NYHA functional classes than the patients without metabolic syndrome. The patients with metabolic syndrome were also significantly more likely to be taking an ACEI, ARB, diuretic, beta-blocker, or CCB than those without metabolic syndrome. As for diabetes treatments, which included insulin, oral therapy, diet control, and other measures, there was no significant difference between the two groups. Conversely, patients without metabolic syndrome were more often never-smokers, and they consumed fewer alcoholic drinks than those with metabolic syndrome.

**Table 1:** Characteristics of patients with and without metabolic syndrome.

**Characteristics**	**All** **(***n*** = 1548)**	**Non-metabolic syndrome** **(***n*** = 351)**	**Metabolic syndrome** **(***n*** = 1,197)**	***p*** **Value**
**Demographics**				
Randomized to MRA, %	781 (50.5)	177 (50.4)	604 (50.5)	0.992
Female	770 (49.7)	178 (50.7)	592 (49.5)	0.08
Age, years	71.2 ± 9.6	73.1 ± 10.3	70.6 ± 9.4	<0.001^*^
**Race**				
White	1212 (78.3)	276 (78.6)	936 (78.2)	0.861
Black	251 (16.2)	54 (21.5)	197 (17.5)	0.139
Other	72 (4.7)	21 (6.0)	51 (4.3)	0.178
**BMI, kg/m^2^**				
<18.5	6 (0.4)	5 (1.4)	1 (0.1)	<0.001^*^
18.5-24.9	143 (9.2)	84 (24.0)	59 (4.9)	<0.001^*^
25-29.9	332 (21.4)	99 (28.3)	233 (19.5)	<0.001^*^
≥30.0	1065 (68.8)	162 (46.3)	903 (75.5)	<0.001^*^
Waist, cm	111.5 ± 17.7	102.8 ± 19.0	114.1 ± 16.4	<0.001^*^
LVEF, %	59.9 ± 7.8	59.0 ± 8.6	60.1 ± 7.5	0.157
Heart rate, b.p.m.	69.0 ± 11.4	68.2 ± 11.4	69.2 ± 11.4	0.148
**Blood pressure, mm/Hg**				
SBP	127.7 ± 16.0	125.0 ± 16.8	128.6 ± 15.7	<0.001^*^
DBP	71.5 ± 11.5	72.2 ± 11.6	71.3 ± 11.4	0.187
**NYHA functional classification, %**				
I & II	1001 (64.7)	246 (70.1)	755 (63.1)	0.016^*^
III & IV	547 (35.3)	105 (29.9)	442 (36.9)	
**Comorbidities**				
Hypertension	1405 (90.8)	246 (70.1)	1159 (96.8)	<0.001^*^
MI	324 (20.9)	36 (10.3)	288 (24.1)	<0.001^*^
PCI	304 (19.6)	30 (8.5)	274 (22.9)	<0.001^*^
CABG	299 (19.3)	18 (5.1)	281 (23.5)	<0.001^*^
Angina pectoris	431 (27.8)	58 (16.5)	373 (31.2)	<0.001^*^
Atrial fibrillation	650 (42.0)	166 (47.3)	484 (40.4)	0.022^*^
Pacemaker	209 (13.5)	47 (13.4)	162 (13.5)	0.945
Implanted cardioverter-defibrillator	35 (2.3)	7 (2.0)	28 (2.3)	0.702
Diabetes mellitus	768 (49.6)	28 (8.0)	740 (61.8)	<0.001^*^
Dyslipidemia	1,076 (69.5)	17 (4.8)	1,059 (88.5)	<0.001^*^
COPD	248 (16.0)	48 (13.7)	200 (16.7)	0.173
Asthma	167 (10.8)	30 (8.5)	137 (11.4)	0.124
Stroke	139 (9.0)	22 (6.3)	117 (9.8)	0.043^*^
Peripheral arterial disease	184 (11.9)	25 (7.1)	159 (13.3)	0.002^*^
Thyroid disease	284 (18.3)	59 (16.8)	225 (18.8)	0.397
Bone fracture	236 (15.2)	53 (15.1)	183 (15.3)	0.917
**Laboratory**				
eGFR, ml/min/1.73 m^2^	64.3 ± 21.7	67.6 ± 24.0	63.3 ± 20.9	0.001^*^
Chlorine, mmol/L	101.4 ± 9.7	102.2 ± 7.1	101.2 ± 10.4	0.072^*^
Potassium, mmol/L	4.2 ± 0.4	4.2 ± 0.4	4.2 ± 0.5	0.970
Glucose, mg/dL	125.7 ± 60.2	97.9 ± 29.7	132.2 ± 67.4	<0.001^*^
BUN, mg/dL	24.1 ± 10.1	22.8 ± 9.1	24.5 ± 10.3	0.011^*^
Hemoglobin, g/dL	12.8 ± 1.7	13.1 ± 1.7	12.8 ± 1.7	0.001^*^
HCT	38.6 ± 4.8	39.3 ± 4.8	38.4 ± 4.8	0.003^*^
TBILI, mg/dL	0.7 ± 0.5	0.8 ± 0.6	0.6 ± 0.4	<0.001^*^
**Medications**				
ACE-I/ARB	1,246 (80.5)	256 (73.2)	990 (82.7)	<0.001^*^
Diuretic	1,390 (89.8)	300 (85.7)	1,090 (91.1)	0.004^*^
Beta blockers	1,220 (78.8)	250 (71.4)	970 (81.0)	<0.001^*^
Calcium channel blocker	611 (39.5)	103 (29.4)	508 (42.4)	<0.001^*^
**Treatment for diabetes mellitus**				
Insulin	366/768 (47.7)	9 (32.1)	357 (48.2)	0.094
Oral therapy	508/768 (66.1)	17 (94.4)	491 (66.4)	0.536
Diet control	364/768 (47.4)	16 (57.1)	348 (47.0)	0.293
Other	11/768 (1.4)	0 (0)	11 (1.5)	1.000
**Lifestyle factors**				
Smoke status, n (%)				
Current	103 (6.7)	24 (6.8)	79 (6.6)	0.878
Past	779 (50.3)	161 (49.2)	618 (55.3)	0.052
Never	586 (37.9)	166 (43.4)	420 (37.6)	0.001^*^
**Alcohol drinks in the past weeks**, ***n*****(%)**				
None	1,158 (74.8)	244 (68.5)	914 (76.4)	0.009^*^
1–4	275 (17.8)	78 (22.2)	197 (16.5)	0.013^*^
5–10	79 (5.1)	21 (6.0)	58 (4.8)	0.394
>11	34 (2.2)	8 (2.3)	26 (2.2)	0.904
**Quality of life**				
Mean KCCQ overall score (+SD)	57.2 ± 23.4	62.8 ± 23.6	55.8 ± 23.1	<0.001^*^
Mean EQ-5D (+SD)	62.8 ± 20.2	66.7 ± 19.3	61.9 ± 20.1	<0.001^*^
**PHQ**				
<10	911 (58.9)	199 (79.3)	712 (71.0)	0.008^*^
≥10	343 (22.2)	52 (20.7)	291 (29.0)	
**Echocardiographic data**				
**Diastolic dysfunction, no. (%)**				
Normal	20 (1.3)	6 (4.8)	14 (3.2)	0.388
Mild	59 (3.8)	16 (12.8)	43 (9.8)	0.329
Moderate	99 (6.4)	16 (12.8)	83 (18.9)	0.116
Severe	60 (3.9)	7 (5.6)	53 (12.0)	0.039^*^

*Values are mean ± SD or %. BMI, Body mass index; LVEF, center ventricular ejection fraction; SBP, Systolic blood pressure; DBP, Diastolic blood pressure; MI, Myocardial infarction; PCI, Percutaneous coronary intervention: CABG, coronary artery bypass graft; eGFR, estimated glomerular filtration rate; BUN, blood urea nitrogen; HCT, hematocrit; ALB, albumin; TBILI, total bilirubin; ACE-I, angiotensin-converting enzyme inhibitors; ARB, angiotensin II receptor blockers; COPD, chronic obstructive pulmonary disease; NYHA, New York Heart Association; NT-proBNP, N-terminal pro-BNP*.

### Differences in the Quality of Life and Echocardiographic Parameters Between the Patients With and Without Metabolic Syndrome

Three self-administered questionnaires (the KCCQ, EQ-5D, and PHQ) were used to assess various health-related aspects of the quality of life. The patients with metabolic syndrome had lower KCCQ and EQ-5D scores and more severe depression compared with the patients without metabolic syndrome. Additionally, the echocardiographic data showed that patients with metabolic syndrome had a higher prevalence of severe diastolic dysfunction than those without metabolic syndrome (Table 1).

### Primary and Secondary Outcomes

**Figure 2:**
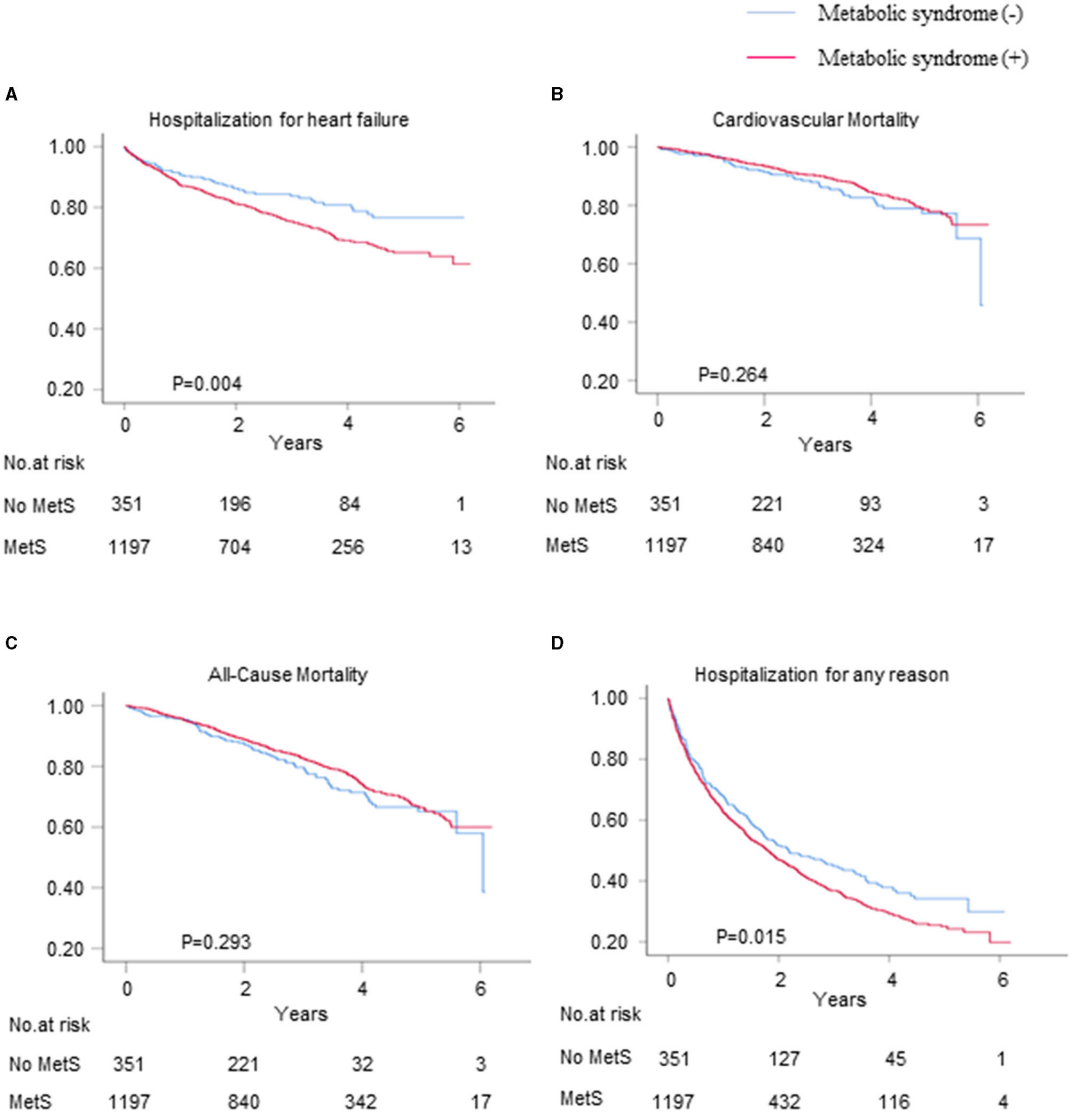
The Kaplan–Meier survival curves and cumulative event rates for the primary and secondary outcomes. The hospitalization rates for **(A)** heart failure (HF), **(B)** cardiovascular (CV) mortality, **(C)** all-cause mortality, and **(D)** hospitalization for any reason.

The mean follow-up duration was 3.3 years. A total of 351 (22.7%) patients were hospitalized for HF within 6 years, and 339 (20.6%) of these patients died, 197 (12.0%) of whom died from CV disease. The HF and all-cause hospitalization rates were higher for the patients with metabolic syndrome than for those without metabolic syndrome (24.6 vs. 16.2%, *p* = 0.001; 62.2 vs. 54.1%, *p* = 0.006, respectively), whereas there were no differences in the CV and all-cause mortality rates between the patients with and without metabolic syndrome (Figures 2B,C). The Kaplan–Meier survival curves and cumulative event rates for HF hospitalization, CV mortality, all-cause mortality, and all-cause hospitalization for the patients with and without metabolic syndrome are shown in Figure 2 and Table 2, respectively. The unadjusted risks of HF hospitalization and all-cause hospitalization were significantly different between the patients with and without metabolic syndrome [unadjusted HF hospitalization HR = 1.52, 95% CI = 1.15–2.02, and *p* = 0.004 (Figure 2A); unadjusted all-cause hospitalization HR = 1.22, 95% CI = 1.04–1.43, and *p* = 0.015 (Figure 2D)]. After performing the multivariable adjustments, the risk of HF hospitalization was significantly higher for the patients with metabolic syndrome than for those without metabolic syndrome (model 1 adjusted HR = 1.60, 95% CI = 1.20–2.13, and *p* = 0.001; model 2 adjusted HR = 1.47, 95% CI = 1.05–2.05, and *p* = 0.025; and model 3 adjusted HR = 1.42, 95% CI = 1.01–2.00, and *p* = 0.042) (Table 2). The risk of all-cause hospitalization was also significantly higher for the patients with metabolic syndrome than for those without metabolic syndrome (model 1 adjusted HR = 1.27, 95% CI = 1.08–1.49, and *p* = 0.004; model 2 adjusted HR = 1.25, 95% CI = 1.03–1.51, and *p* = 0.023; and model 3 adjusted HR = 1.27, 95% CI = 1.04–1.54, and *p* = 0.017) (Table 2). Regardless of whether adjustments were made, however, there were no differences in the CV mortality between the patients with and without metabolic syndrome (unadjusted HR = 0.83, 95% CI = 0.60–1.15, and *p* = 0.264; model 1 adjusted HR = 0.95, 95% CI = 0.68–1.32, and *p* = 0.765; model 2 adjusted HR = 1.04, 95% CI = 0.69–1.57, and *p* = 0.844; and model 3 adjusted HR = 1.02, 95% CI = 0.67–1.55, and *p* = 0.934), nor were there differences in the all-cause mortality between the patients with and without metabolic syndrome (unadjusted HR = 0.87, 95% CI = 0.68–1.12, and *p* = 0.293; model 1 adjusted HR = 1.01, 95% CI = 0.78–1.30, and *p* = 0.938; model 2 adjusted HR = 1.04, 95% CI = 0.76-1.42, and *p* = 0.799; and model 3 adjusted HR = 1.06, 95% CI = 0.77–1.46, and *p* = 0.716) (Table 2).

**Table 2:** The risk of HF hospitalization, cardiovascular mortality, all-cause mortality and all-cause hospitalization in HFpEF patients with and without metabolic syndrome.

**Characteristics**	**Non-metabolic syndrome** **(***n*** = 351)**	**Metabolic syndrome** **(***n*** = 1,197)**	***p*** **Value**
**Hospitalization for HF**			
Cases/*n*	57/351	294/1197	
Unadjusted HR (95% CI)	1.00 (ref)	1.52 (1.15–2.02)	0.004
Model 1: adjusted HR (95% CI)	1.00 (ref)	1.60 (1.20–2.13)	0.001
Model 2: adjusted HR (95% CI)	1.00 (ref)	1.47(1.05–2.05)	0.025
Model 3: adjusted HR (95% CI)	1.00 (ref)	1.42(1.01–2.00)	0.042
**Cardiovascular mortality**			
Cases/*n*	49/351	148/1197	
Unadjusted HR (95% CI)	1.00 (ref)	0.83 (0.60–1.15)	0.264
Model 1: adjusted HR (95% CI)	1.00 (ref)	0.95 (0.68–1.32)	0.765
Model 2: adjusted HR (95% CI)	1.00 (ref)	1.04 (0.69–1.57)	0.844
Model 3: adjusted HR (95% CI)	1.00 (ref)	1.02 (0.67–1.55)	0.934
**All-cause mortality**			
Cases/*n*	81/351	258/1197	
Unadjusted HR (95% CI)	1.00 (ref)	0.87 (0.68–1.12)	0.293
Model 1: adjusted HR (95% CI)	1.00 (ref)	1.01 (0.78–1.30)	0.938
Model 2: adjusted HR (95% CI)	1.00 (ref)	1.04 (0.76–1.42)	0.799
Model 3: adjusted HR (95% CI)	1.00 (ref)	1.06 (0.77–1.46)	0.716
**All-cause hospitalization**			
Cases/*n*	190/351	745/1197	
Unadjusted HR (95% CI)	1.00 (ref)	1.22 (1.04–1.43)	0.015
Model 1: adjusted HR (95% CI)	1.00 (ref)	1.27 (1.08–1.49)	0.004
Model 2: adjusted HR (95% CI)	1.00 (ref)	1.25 (1.03–1.51)	0.023
Model 3: adjusted HR (95% CI)	1.00 (ref)	1.27 (1.04–1.54)	0.017

*In model 1, the following parameters were adjusted: sex, age, race, smoking status and alcohol. In model 2, the following parameters were adjusted: sex, age, race, smoking status and alcohol, NYHA functional class, MI, angina pectoris, atrial fibrillation, stroke, peripheral arterial disease, implanted cardioverter defibrillator, implanted pacemaker, chronic obstructive pulmonary disease, treatment by percutaneous coronary intervention or coronary artery bypass graft surgery, eGFR, urea nitrogen, hematocrit, potassium, chlorine, TBILI and randomization arm (spironolactone or placebo). In model 3, the following parameters were adjusted: sex, age, race, smoking status and alcohol, NYHA functional class, MI, angina pectoris, atrial fibrillation, stroke, peripheral arterial disease, implanted cardioverter defibrillator, implanted pacemaker, chronic obstructive pulmonary disease, treatment by percutaneous coronary intervention or coronary artery bypass graft surgery, eGFR, urea nitrogen, hematocrit, potassium, chlorine, TBILI and randomization arm (spironolactone or placebo), use of angiotensin II receptor blockers or angiotensin-converting enzyme inhibitors, calciumchannel blockers, beta blockers and diuretics*.

**Figure 3:**
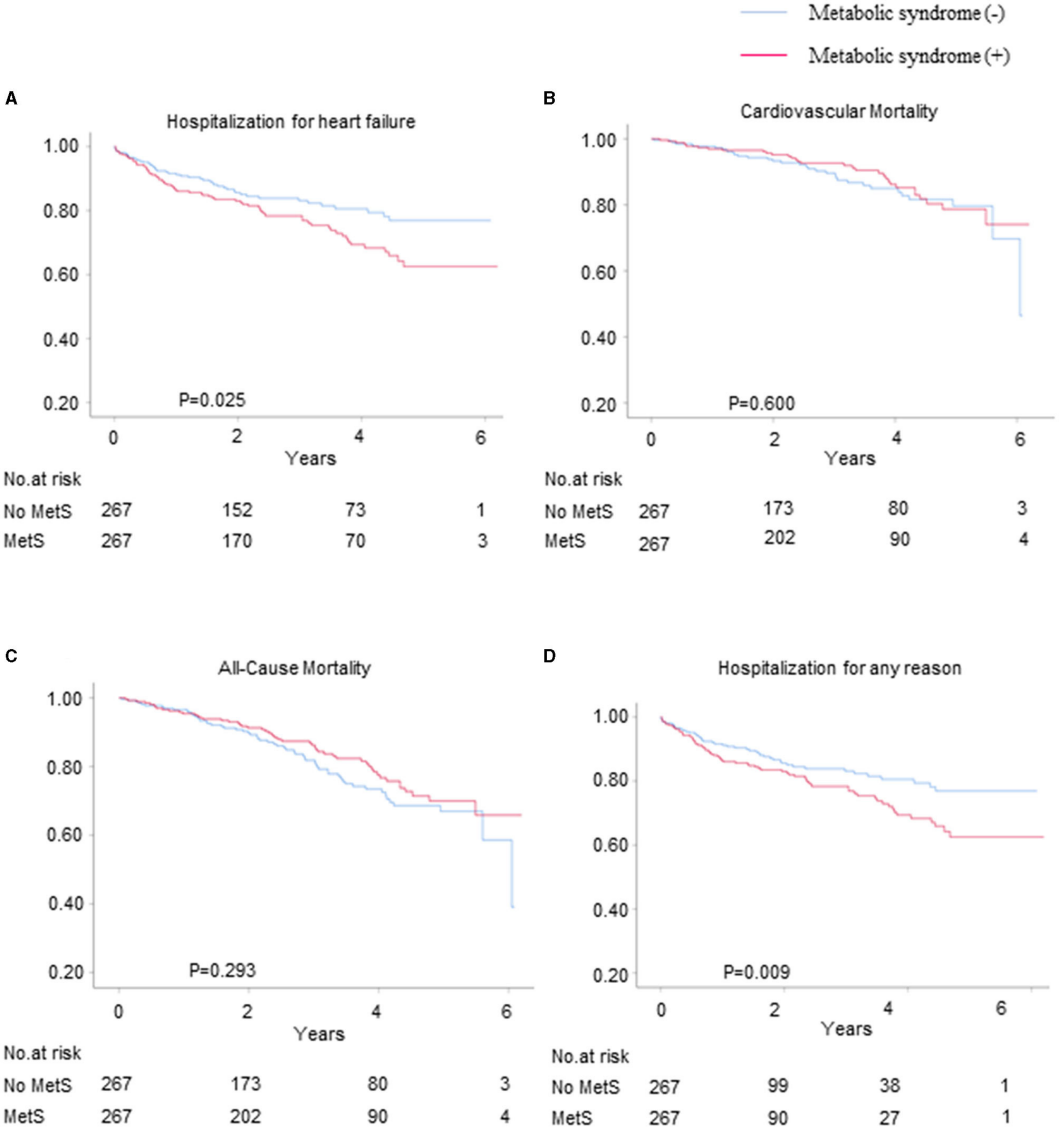
The Kaplan–Meier survival curves for the primary and secondary outcomes for the propensity score-matched patients with and without metabolic syndrome. The hospitalization rates for **(A)** HF, **(B)** CV mortality, **(C)** all-cause mortality, and **(D)** hospitalization for any reason.

We used propensity score matching for the sensitivity analyses to verify the associations between metabolic syndrome and the risks of HF hospitalization, CV mortality, all-cause mortality, and all-cause hospitalization for patients with HFpEF. Among the propensity score-matched patients (*n* = 267), the risks of HF hospitalization and all-cause hospitalization were significantly higher for those with metabolic syndrome than for those without metabolic syndrome (HR = 1.53, 95% CI = 1.05–2.23, and *p* = 0.025 and HR = 1.34, 95% CI = 1.08–1.67, and *p* = 0.009, respectively) (Figures 3A,D), whereas there were no differences between the patients with and without metabolic syndrome in terms of the risks of CV mortality and all-cause mortality (Figures 3B,C).

**Figure 4:**
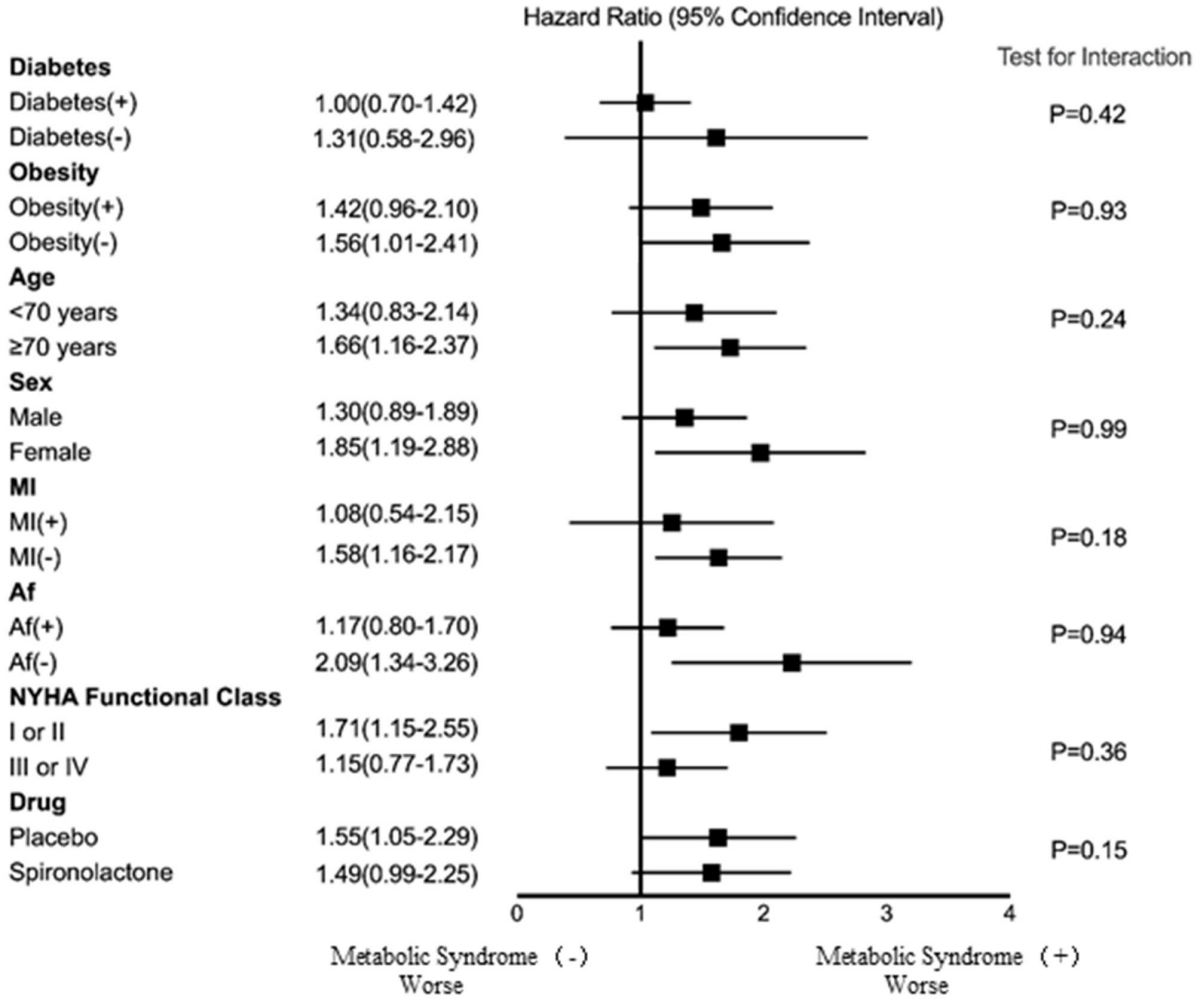
The association between metabolic syndrome and risk of HF hospitalization for the different subgroups. Af, atrial fibrillation; MI, myocardial infarction; NYHA, New York Heart Association.

Figure 4 shows the association between the patients with metabolic syndrome and the risk of HF hospitalization in the different subgroups based on age, sex, MI, AF, NYHA functional class, obesity, diabetes, and treatment arm. Although not all the subgroups showed a statistically significant association with the risk of HF hospitalization, the results indicate that the risk of HF hospitalization for the older, female, no MI, no AF, lower NYHA functional class, and placebo treatment arm subgroups were higher among the patients with metabolic syndrome than among those without metabolic syndrome. However, there were no interactions between metabolic syndrome and age, sex, MI, NYHA functional class, AF, obesity, diabetes, or spironolactone use.

## Discussion

The results of this analysis highlight the clinical importance of metabolic syndrome in stable individuals with HFpEF. The patients with HFpEF and metabolic syndrome in this study had more CV comorbidities, worse renal function, worse quality of life, took more antihypertensive medications, and they suffered from more severe diastolic dysfunction than those without metabolic syndrome. The current study further assessed the association between metabolic syndrome and the risk of hospitalization due to HF for patients with HFpEF. The results demonstrate that the risk of rehospitalization for HF or any other reason was significantly higher for patients with metabolic syndrome than for those without metabolic syndrome. However, there were no differences in CV mortality and all-cause mortality between the two groups. Importantly, the metabolic syndrome in patients with HFpEF was independently associated with an increased risk of hospitalization for the management of HF or any other reason after adjusting for confounding variables. Notably, an association between metabolic syndrome and an increased risk of HF hospitalization was observed in all the clinically relevant subgroups.

The incidence of metabolic syndrome is high in patients with HF (11), and the results of the present study demonstrate that the majority (77.3%) of TOPCAT participants in America had metabolic syndrome. Insulin resistance and central obesity are postulated to be involved in metabolic syndrome, and both lead to glucose intolerance and dysglycemia (12). Specifically, insulin resistance progresses toward hyperinsulinemia and hyperglycemia, thus triggering peripheral vasoconstriction and sodium retention (13, 14). Moreover, adipocytes secrete mediators of insulin resistance, namely, TNF-β, leptin, adiponectin, and resistin (12).

Abdominal obesity is known to be a major risk factor for CV disease, while peripheral adipose tissue confers protective effects, which leads to the obesity “paradox” in HF (13). A recent study showed that even in non-obese individuals, abdominal fat deposition is associated with several adverse cardiac functions that are independent of BMI (15), suggesting that not only the amount but also the location of adipose tissue may be important in patients with HFpEF. Another study showed that metabolic syndrome, defined by the International Diabetes Federation criteria that include obesity, is associated with improved survival in patients with HF (14). The authors found that patients with HF having metabolic syndrome but without diabetes mellitus showed better survival compared to patients with HF having diabetes mellitus but not a metabolic syndrome or patients with HF and without diabetes mellitus or metabolic syndrome, which is the group that showed the worst survival rate. These observations might suggest that insulin resistance affects the prognosis of patients with HF independently from obesity, which is consistent with previous studies that showed that non-obese individuals with metabolic syndrome are at an increased risk of developing HF compared to obese individuals without metabolic syndrome. As there are few studies that have assessed the prognostic implications of adipose tissue increases in underweight or normal-weight patients with HF, future research is necessary on this topic.

With regards to metabolic syndrome, HFpEF is generally considered to be an inflammatory disease (16). This may lead to a higher prevalence of metabolic syndrome in the HFpEF population than in the general population. In addition, a previous cohort study showed that the prevalence of metabolic syndrome among Hispanic patients with HF was 78.8%, followed by 69.5% for white patients, and 60.9% for black patients (17). Moreover, age, female gender, hypertension, diabetes, obesity, metabolic syndrome, renal dysfunction, high waist-to-hip ratio, and physical inactivity were identified as classical risk factors for the development of HFpEF, thus it is not surprising that 85% of patients with HFpEF have metabolic syndrome (16). For this reason, it is necessary to determine the impact of metabolic syndrome on HFpEF.

The patients with HFpEF having metabolic syndrome in the present study were characterized by younger age, a higher prevalence of coronary artery disease, lower NYHA class, higher prevalence of current smoking and alcohol drinking habits, and a higher likelihood of taking medications, such as ACEIs/ARBs, beta-blockers, diuretics, or CCBs. These clinical features are consistent with the results from a Japanese HF cohort (5), in which the authors suggested that comorbidity-specific treatments and multifactorial lifestyle modification interventions are likely the most effective methods for reducing the burden of HFpEF. In addition, our results show that patients with metabolic syndrome have a lower quality of life and worse depression, suggesting a critical need for adjusting treatment and management strategies for patients with HFpEF having metabolic syndrome to improve their quality of life.

The echocardiographic findings in the present study show that the patients with metabolic syndrome were characterized by worse diastolic function compared to those without metabolic syndrome. Notably, the echocardiographic data from the Phosphodiesterase-5 Inhibition to Improve Clinical Status and Exercise Capacity in Diastolic Heart Failure (RELAX) and the Irbesartan in Heart Failure with Preserved Systolic Function (I-PRESERVE) trials suggested that diabetes mellitus was associated with more severe left ventricular (LV) diastolic dysfunction and hypertrophy and underlying metabolic derangements and systemic inflammation may account for this phenomenon (18, 19). Specifically, the occurrence of insulin resistance in patients with metabolic syndrome may lead to increased uptake of free fatty acids by cardiomyocytes, resulting in mitochondrial dysfunction, production of toxic lipid intermediates, and increased reactive oxygen species (20, 21). Moreover, adipocytes secrete proinflammatory cytokines, and advanced glycation end-products induced by hyperglycemia damage microvascular function and accelerate endothelial dysfunction (20–23). All these pathological processes may contribute to LV diastolic function deterioration.

The results of the present study show that the risk of HF hospitalization was significantly higher in patients with metabolic syndrome than in those without metabolic syndrome. The consensus is that increased neurohumoral activation and changes in sodium metabolism in patients with metabolic syndrome may precede vascular congestion, cardiorenal syndrome, and a decreased diuretic response (21, 24). Hospitalization for management of HF would subsequently manifest in patients with metabolic syndrome due to volume overload.

Interestingly, the results of our analysis of the association between metabolic syndrome and mortality differ from those of the previous studies (25). In a retrospective study of a cohort of patients admitted with HF, Hassan et al. reported a lower mortality rate for the patients with HF having metabolic syndrome compared to those without metabolic syndrome (17). On the contrary, Tamariz et al. reported a mortality rate of 24% for patients with metabolic syndrome compared to 16% for those without metabolic syndrome in a prospective study of patients who had an LVEF >40% at 2.6 years of follow-up (26). Our results demonstrate that both CV mortality and all-cause mortality were not significantly affected by metabolic syndrome for the patients with HFpEF. Overall, our understanding of the relationship between metabolic syndrome and the outcomes of patients with HFpEF is still limited. Therefore, long-term follow-up studies are needed to assess mortality rates after specific interventions.

Although previous studies have demonstrated that spironolactone is beneficial for managing HF and metabolic syndrome (27–29), spironolactone therapy did not significantly improve the adverse outcomes of the patients with HFpEF having metabolic syndrome in the present study. Recent studies have shown that activation of the renin-angiotensin system does not reduce the mortality of patients with HFpEF (21). Remarkably, however, clinical trials with sodium-glucose cotransporter-2 (SGLT2) inhibitors have shown an improvement in the prognosis of HF (30). Theoretically, SGLT2 inhibitors exert cardioprotective effects by inducing a state of fasting mimicry and suppressing SIRT1/AMPK signaling and autophagy, both of which decrease inflammation markers (31). Bode et al. (32) showed in a rat model of HFpEF related to metabolic syndrome that rats that received the SGLT2 inhibitor Sotagliflozin exhibited improved metabolic left atrial remodeling and arrhythmia characteristics. SGLT2 inhibitors may also be beneficial for the metabolism of ketones in the heart, thereby reducing oxygen consumption and free radical production (33). In addition, a previous meta-analysis showed that GLP-1R agonists are effective for improving LV diastolic function in the treatment of patients with HFpEF (34). Therefore, SGLT2 inhibitors and GLP-1R agonists represent a promising approach to treating HFpEF and metabolic syndrome.

## Limitations

This was a *post-hoc*, exploratory analysis of the TOPCAT trial; therefore, randomization may break, and residual and uncontrolled confounding may still be present. In addition, the data from the clinical trials may not be representative of real-world HFpEF populations. Furthermore, the original data do not specify separate NYHA1 or NYHA2 values; therefore, it was impossible to analyze the NYHA1 and NYHA2 results separately. Finally, it may be difficult to eliminate reverse outcomes completely.

## Conclusion

The patients with HFpEF having metabolic syndrome in the TOPCAT cohort were younger and had more severe depression, more comorbidities, and worse health status than those without metabolic syndrome. Although there were no significant differences in mortality between the patients with and without metabolic syndrome, the metabolic syndrome was independently associated with an increased risk of HF hospitalization and all-cause hospitalization during the follow-ups.

## Data Availability Statement

The original contributions presented in the study are included in the article/Supplementary Material, further inquiries can be directed to the corresponding authors.

## Author Contributions

YW and ST defined the study theme and methods. YZ, LF, and JS analyzed the data. YZ wrote the paper. ZX, ZZ, ST, and SZ edited the paper. All authors read and approved the final manuscript.

## Funding

This research was supported by the National Natural Science Foundation of China (Grant number 81801394, awarded to ST) and the Natural Science Foundation of Hunan Province (Grant number 2019JJ50878, awarded to ST).

## Conflict of Interest

The authors declare that the research was conducted in the absence of any commercial or financial relationships that could be construed as a potential conflict of interest.

## Publisher's Note

All claims expressed in this article are solely those of the authors and do not necessarily represent those of their affiliated organizations, or those of the publisher, the editors and the reviewers. Any product that may be evaluated in this article, or claim that may be made by its manufacturer, is not guaranteed or endorsed by the publisher.
