# Supplementary material for: Association Between Metabolic Syndrome and an Increased Risk of Hospitalization for Heart Failure in Population of HFpEF
Source: Front Cardiovasc Med. 2021 Sep 14;8:698117. doi: 10.3389/fcvm.2021.698117 (PMC8476762; doi:10.3389/fcvm.2021.698117)
Supplement: Supplementary file 1 [file Data_Sheet_1.docx]

| **Supplemental Table.** Baseline Characteristics of Study Patients With and Without Metabolic Syndrome After Matching | | | | |
| --- | --- | --- | --- | --- |
|  | **All (n=534)** | **Non-metabolic syndrome**  **(n=267)** | **metabolic**  **syndrome**  **(n=267)** | **p Value** |
| Demographics |  |  |  |  |
| Randomized to MRA, % | 269(50.4) | 135(50.6) | 134(50.2) | 0.931 |
| Female | 275(51.5) | 137(51.3) | 138(51.7) | 0.931 |
| Age, years | 72.8±10.1 | 73.5±10.6 | 72.12±9.6 | 0.040* |
| Race |  |  |  |  |
| White | 413(77.3) | 211(79.0) | 202(75.7) | 0.352 |
| Black | 94(17.6) | 40(15.0) | 54(20.2) | 0.112 |
| Other | 27(5.1) | 16(6.0) | 11(4.1) | 0.323 |
| BMI, kg/m^2^ |  |  |  |  |
| <18.5 | 4(0.7) | 4(1.5) | 0(0) | 0.124 |
| 18.5-24.9 | 73(13.7) | 63(23.6) | 10(3.7) | <0.001* |
| 25-29.9 | 126(24.0) | 71(27.0) | 55(20.9) | 0.102 |
| >=30.0 | 331(62.0) | 129(48.3) | 202(75.7) | <0.001* |
| Waist, cm | 108.5±18.2 | 103.6±19.7 | 113.4±15.2 | <0.001* |
| LVEF, % | 59.9±7.8 | 60.4±7.0 | 60.0±7.7 | 0.547 |
| Heart rate, b.p.m. | 68.7±11.2 | 67.7±11.1 | 69.7±11.3 | 0.269 |
| Blood pressure, mm/Hg |  |  |  |  |
| SBP | 125.8±15.5 | 123.9±15.6 | 127.8±15.2 | 0.907 |
| DBP | 70.9±11.3 | 71.0±11.6 | 70.9±11.1 | 0.445 |
| NYHA functional classification, % |  |  |  |  |
| I & II | 345(64.6) | 179(67.0) | 166(63.1) | 0.239 |
| III & IV | 189(35.4) | 88(33.0) | 101(37.8) |  |
| Comorbidities |  |  |  |  |
| Hypertension | 440(82.4) | 184(68.9) | 256(95.9) | <0.001* |
| MI | 72(13.5) | 28(10.5) | 44(16.5) | 0.043* |
| PCI | 62(11.6) | 25(9.4) | 37(13.9) | 0.105 |
| CABG | 55(10.3) | 16(6.0) | 39(14.6) | 0.001* |
| Angina pectoris | 113(21.2) | 48(18.0) | 65(24.3) | 0.072 |
| Atrial fibrillation | 271(50.7) | 141(52.8) | 130(47.8) | 0.341 |
| Pacemaker | 77(14.4) | 41(15.4) | 36(13.5) | 0.538 |
| Implanted cardioverter-defibrillator | 12(2.2) | 7(2.6) | 5(1.9) | 0.559 |
| Diabetes mellitus | 163(30.5) | 22(8.2) | 141(52.8) | <0.001* |
| Dyslipidemia | 249(46.6) | 16(6.0) | 233(87.3) | <0.001* |
| COPD | 88(16.5) | 37(13.9) | 51(19.1) | 0.102 |
| Asthma | 65(12.2) | 26(9.7) | 39(14.6) | 0.085 |
| Stroke | 43(8.1) | 15(5.6) | 28(10.5) | 0.039* |
| Peripheral arterial disease | 48(9.0) | 21(7.9) | 27(10.1) | 0.364 |
| Thyroid disease | 114(21.3) | 50(18.7) | 64(24.0) | 0.139 |
| Bone fracture | 105(19.7) | 48(18.0) | 57(21.3) | 0.327 |
| Laboratory |  |  |  |  |
| eGFR, ml/min/1.73 m^2^ | 66.1±22.5 | 66.8±24.5 | 65.3±20.3 | 0.432 |
| Chlorine, mmol/L | 102.4±6.1 | 102.8±3.6 | 102.0±7.7 | 0.520 |
| Potassium, mmol/L | 4.1±0.4 | 4.1±0.4 | 4.2±0.5 | 0.731 |
| Glucose, mg/dL | 115.0±49.4 | 93.1±47.6 | 129.7±59.5 | 0.001* |
| BUN, mg/dL | 24.3±11.9 | 24.2±12.2 | 24.3±11.6 | 0.785 |
| Hemoglobin, g/dL | 13.1±1.6 | 13.1±1.6 | 13.0±1.5 | 0.346 |
| HCT | 39.1±4.6 | 39.1±4.8 | 39.0±4.3 | 0.082 |
| TBILI, mg/dL | 0.7±0.4 | 0.7±0.4 | 0.7±0.4 | 0.521 |
| Medications |  |  |  |  |
| ACE-I/ARB | 374(70.0) | 184(68.9) | 190(71.2) | 0.571 |
| Diuretic | 462(86.5) | 227(85.0) | 235(88.0) | 0.311 |
| Beta blockers | 413(77.3) | 199(74.5) | 214(80.1) | 0.121 |
| Calcium channel blocker | 182(34.1) | 81(30.3) | 101(37.8) | 0.068 |
| Treatment for diabetes mellitus |  |  |  |  |
| Insulin | 68/163(41.7) | 7(31.8) | 61(43.3) | 0.311 |
| Oral therapy | 106/163(65.0) | 14(63.6) | 92(65.2) | 0.883 |
| Diet control | 87/163(47.4) | 14(63.6) | 73(51.8) | 0.300 |
| Other | 0/163(0) | 0(0) | 0(0) | 1.000 |
| Lifestyle factors |  |  |  |  |
| Smoke status, n (%) |  |  |  |  |
| Current | 34(6.4) | 13(4.9) | 21(7.9) | 0.156 |
| Past | 259(51.8) | 127(50.0) | 132(53.7) | 0.413 |
| Never | 254(48.8) | 140(55.1) | 114(42.7) | 0.005* |
| Alcohol drinks in the past weeks, n (%) |  |  |  |  |
| None | 365(68.4) | 174(65.2) | 191(71.5) | 0.114 |
| 1–4 | 117(21.9) | 67(25.1) | 50(18.7) | 0.075 |
| 5–10 | 32(6.0) | 18(6.7) | 14(5.2) | 0.466 |
| >11 | 20(3.7) | 8(3.0) | 12(4.5) | 0.362 |
| Quality of life |  |  |  |  |
| Mean KCCQ overall score (+SD) | 61.2±22.6 | 64.2±22.9 | 58.3±22.0 | 0.122 |
| Mean EQ-5D (+SD) | 64.6±19.8 | 66.4±18.9 | 62.9±20.6 | 0.284 |
| PHQ |  |  |  |  |
| <10 | 383(77.5) | 192(79.0) | 191(76.1) | 0.437 |
| >=10 | 111(22.5) | 51(21.0) | 60(23.9) |  |
| Echocardiographic Data |  |  |  |  |
| Diastolic dysfunction, no. (%) |  |  |  |  |
| Normal | 12(12.4) | 4(8.3) | 8(16.3) | 0.232 |
| Mild | 27(27.8) | 13(27.1) | 14(28.6) | 0.870 |
| Moderate | 15(15.5) | 9(18.8) | 6(12.2) | 0.376 |
| Severe | 43(44.3) | 22(45.8) | 21(42.9) | 0.768 |
| Values are mean ± SD or %.  BMI: Body mass index; LVEF: left ventricular ejection fraction; SBP: Systolic blood pressure; DBP: Diastolic blood pressure; MI; Myocardial infarction; PCI: Percutaneous coronary intervention: CABG: coronary artery bypass graft; eGFR: estimated glomerular filtration rate; BUN: blood urea nitrogen; HCT: hematocrit; ALB: albumin; TBILI: total bilirubin; ACE-I: angiotensin-converting enzyme inhibitors; ARB: angiotensin II receptor blockers; COPD: chronic obstructive pulmonary disease; NYHA: New York Heart Association; NT-proBNP: N-terminal pro-BNP | | | | |
